# Understanding the Pathogenesis of Angelman Syndrome through Animal Models

**DOI:** 10.1155/2012/710943

**Published:** 2012-07-08

**Authors:** Nihar Ranjan Jana

**Affiliations:** Cellular and Molecular Neuroscience Laboratory, National Brain Research Centre, Manesar, Gurgaon 122 050, India

## Abstract

Angelman syndrome (AS) is a neurodevelopmental disorder characterized by severe mental retardation, lack of speech, ataxia, susceptibility to seizures, and unique behavioral features such as easily provoked smiling and laughter and autistic features. The disease is primarily caused by deletion or loss-of-function mutations of the maternally inherited *UBE3A* gene located within chromosome 15q11-q13. The *UBE3A* gene encodes a 100 kDa protein that functions as ubiquitin ligase and transcriptional coactivator. Emerging evidence now indicates that UBE3A plays a very important role in synaptic function and in regulation of activity-dependent synaptic plasticity. A number of animal models for AS have been generated to understand the disease pathogenesis. The most widely used model is the *UBE3A*-maternal-deficient mouse that recapitulates most of the essential features of AS including cognitive and motor abnormalities. This paper mainly discusses various animal models of AS and how these models provide fundamental insight into understanding the disease biology for potential therapeutic intervention.

## 1. Introduction

In 1965, Dr. Harry Angelman first described that 3 of his child patients showed severe mental retardation, jerky movements, excessive laughter, and abnormal physical development. He called them “puppet children” because they resembled puppets with their flat heads. All three showed typically common behavioural features that led him to suggest the possibility of a distinct syndrome. Later the disease was named as Angelman syndrome (AS). Children with AS show developmental delay, lack of speech, ataxia, learning disability, flat occiput, seizures, tongue protrusion, and uncontrollable laughter. Individuals suffering from this disorder show hyperactivity and restless behaviour, wide gait, hypotonia, microcephaly, widely spaced teeth, abnormal EEG patterns, hypopigmentation with blond hair and light eyes, love for water, and dysmorphic features like prominent chin and deep set eyes [1, 2]. Intellectual disability has been described as a feature of AS in almost all studies including the first report by Dr. Angelman [2, 3]. The severity of intellectual disability varies amongst the individuals. Many cases of AS seem to associate with autism [4, 5], which is characterized by reduced social interaction, lack of communication, and stereotypic behavior [6]. 

## 2. Genetics of AS

The cause behind AS remained unknown until the late eighties. High resolution chromosome banding technique revealed that one of the AS patient had a deletion of chromosome 15q11-12 [7]. This was confirmed when a group of children with severe mental retardation, ataxia, and seizures were shown to have a deletion in the proximal long arm of chromosome 15 [7, 8]. Although this deletion had already been reported in Prader-Willi Syndrome (PWS) earlier [9, 10], these children showed features suggestive of AS rather than PWS. The difference in the manifestation of the two syndromes proposed that the genes responsible for both syndromes might be closely associated but definitely distinct. Later AS was mapped within the 15q11-13 region of the chromosome [11]. Another major breakthrough came when it was found through RFLP (restriction fragment length polymorphisms) that deletion in the maternal copy of the chromosome led to AS in contrast to the paternal inheritance of PWS [12, 13]. While 60–70% of the AS cases showed large (3-4 Mb) de novo deletions in chromosome 15 [14], less than 5% of cases showed uniparental paternal disomy (UPD) [15, 16], and 2-3% cases occurred due to imprinting defects [1, 2]. The remaining 25% of the cases had unknown origin but few of them were observed to be familial [17]. A recent clinical study with 160 AS patients suggested that characteristic EEG patterns could be an important biomarker in AS and might predict the underlying genetic cause [18].

**Figure 1:**
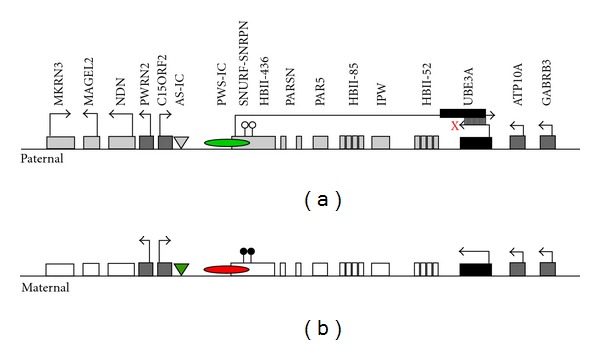
Imprinting map of the human chromosome 15q11-13 region around AS imprinting centre (AS-IC). Paternal and maternal chromosome 15q11-13 regions around AS-IC and PWS-IC are represented in (a) and (b), respectively. Paternally expressed genes (gray boxes), maternally expressed genes (black boxes), maternally repressed genes (white boxes), and biallelically expressed genes (dark gray boxes) are represented with arrows marking transcription start sites. Right arrow indicates gene transcription on “+” strand, whereas left arrow indicates gene transcription on “−” strand. AS-IC (triangle) and PWS-IC (ellipse) are shaded depending on histone modification in the area. AS-IC is dormant (gray triangle) on paternal chromosome, whereas on the maternal chromosome it is acetylated and methylated at H3-lys4 (green triangle), thus active. PWS-IC is active on paternal chromosome (green ellipse) since it is also acetylated and methylated at H3-lys4. However, PWS-IC at the maternal chromosome is methylated at H3-lys9 and repressed (red ellipse). Differentially CpG methylated region (DMR1) in SNRPN exon 1 overlaps with PWS-IC partially. Note that DMR1 on maternal but not paternal chromosome is methylated (black pin). UBE3A-ATS (antisense transcript) originating upstream of SNRPN can either be a degradable complex with *UBE3A* transcript or prevent the extension of *UBE3A* transcript (collision or upstream histone modifications represented by “X”).

In 1994, two candidate genes were mapped to the AS critical region, E6-AP (E6 associated protein encoded by the *UBE3A* gene) and PAR-2 for Prader-Willi/Angelman region-gene-2. Soon mutations in the *UBE3A* gene were found in around 5–10% cases of AS [19, 20]. Discovery of point mutations in *UBE3A* gene strongly implicated *UBE3A* as the gene responsible for AS [20, 21]. Although we cannot dismiss the involvement of other genes in AS, *UBE3A* is the only gene to date whose dysfunction is sufficient to manifest the AS phenotype in number of animal models. It is also important to mention that along with various other chromosomal aberrations identified in autism, maternal deletions and duplication in the proximal region of 15q (region deleted in most cases of AS) are a common cause of autism [22, 23]. *UBE3A* gene was suggested as a strong candidate for autism because of its imprinted nature and maternal dominance [22, 24]. A whole genome wide screening for copy number variation revealed *UBE3A* as one of the affected genomic loci in autism [25]. A map of the maternal and paternal human chromosome region 15q11-13 containing multiple genes is shown in Figure 1.

## 3. UBE3A/E6-AP Protein

*UBE3A *gene is located within the q11-q13 region on chromosome 15 in humans while it is found on the proximal region of chromosome 7 in mice [26]. It encodes a 100 kDa protein known earlier as E6-AP (E6 associated protein) [27, 28]. *UBE3A *gene encodes five mRNA subtypes generated by alternate splicing that give rise three protein isoforms [29]. The functional significance of different isoforms is still unclear. The murine homolog is slightly longer with 885 amino acids. There is about 99% similarity in human and murine E6-AP/UBE3A protein [27]. E6-AP/UBE3A belongs to the HECT (homologous to E6-AP C-terminus) domain family of E3 ubiquitin ligases in the ubiquitin proteasome system (UPS). These proteins exit with large diversity and promote degradation of short lived or abnormal proteins by transferring multiubiquitin molecules to them as a degradation signal [30]. The members of the HECT family share a ~350-residue conserved C-terminal region called the HECT domain [31, 32]. UBE3A is the founding member of the family, discovered based on its interaction with viral E6 oncoprotein to target p53 for proteasomal degradation in cells infected with human papilloma virus (HPV) [28]. 

UBE3A is also demonstrated to act as a transcriptional coactivator of steroid hormone receptors [45–47]. UBE3A is shown to interact with number of cellular proteins that indicate its involvement in multiple cellular function including cell cycle regulation [48–52], synaptic function and plasticity [53–58], and cellular protein quality control [59–61]. A list of identified substrates and possible cellular function of UBE3A is shown in Table 1.

## 4. Mouse Models of AS

 The first attempt to model AS was made in 1992 [62]. This group successfully made a model for PWS with maternal duplication in the central region of chromosome 7 but failed to make the same for AS with paternal duplication. While the imprinting was expected in the central region on the mouse chromosome 7, (which was considered homologous to the human region 15q11-13 deleted in PWS/AS) the actual imprinting seen in the partial UPD mice was more proximal on the chromosome. Hence this was not considered an appropriate model for AS. A few years later, based on detailed investigation by the same group, this mouse model was strongly put forward as a model for AS [40]. Detailed study in this model suggested that the imprinted proximal region earlier identified in fact should be included in the putative PWS/AS segment. The mouse model showed various features like gait ataxia, abnormal limb clasping, startle response, and hyperactivity. The cerebral hemispheres did not show any gross abnormality or cell loss but cortical thinning was noticed. Reduction in the size of the cerebellum was also shown. Abnormal EEG, a typical feature of AS [63, 64], is also recorded in these mice. Soon after the discovery of *UBE3A* mutations in AS individuals [65, 66], this model was further characterized for the expression of *UBE3A,* and found that the expression of this gene was absent in the hippocampus, cerebellar Purkinje cells, and olfactory bulb (mitral-cell layer) of the mice [67]. This shows that majority of the expression observed in these areas is from the maternal allele. Using RNA *in situ* hybridization, it was shown that the cortex showed reduced levels of the *UBE3A* transcript, while there was no change in the anterior commissure and optic chiasm. This suggests that the *UBE3A* gene has varied expression in different region of the brain. Areas like the cerebral cortex, which show reduced expression, have slight predominance of maternal expression, while optic chiasm and anterior commissure have equal expression from both the maternal and paternal alleles. Imprinting in the AS brain was reported around the same time [24, 68], but Albrecht et al. [67] failed to detect imprinting in the whole mouse brain. Therefore, they looked into different parts of the brain and concluded that *UBE3A* is imprinted only in certain areas of the brain. The absence of *UBE3A* had no effect on the number of Purkinje cells or the overall cytoarchitecture of the brain in UPD mice. 

The most widely used model of AS is the *UBE3A* knockout mice. This mouse was generated by a deletion mutation in exon 2 of *UBE3A* gene thereby inhibiting the formation of a functionally active protein [33]. Mice generated were termed wild-type *UBE*3*A* ^m+/p+^, heterozygous *UBE*3*A* ^m−/p+^- or *UBE*3*A* ^m+/p−^ (depending upon the parental inheritance), and homozygous *UBE*3*A* ^m−/p−^ (null) for the mutation. The maternal deficient heterozygous mice *UBE*3*A* ^m−/p+^ exhibited reduced brain weight, ataxia, motor impairment, and abnormal EEG pattern. Around 20–30% of maternal-deficient and null mice exhibited audiogenic seizures. The maternal deficient mice also showed context-dependent learning and memory impairment and deficits in hippocampal long-term potentiation. UBE3A expression was imprinted in hippocampus and cerebellar Purkinje cells, and p53 level was increased in the Purkinje cells of *UBE*3*A* ^m−/p+^ mice [33]. This genetic model successfully captured many of the classical features associated with AS and provides a tool to discover molecules and pathways affected by the absence of UBE3A, mainly the ones responsible for cognitive and motor function. 

Detailed immunohistochemical and immunoblot analysis later revealed that *UBE3A* in these mice is imprinted throughout the brain. Various areas of the brain like cortex, striatum, midbrain, and hypothalamus in addition to hippocampus, cerebellum, and olfactory bulbs showed predominant expression from the maternal copy of the chromosome [34, 35, 69]. It was reported that along with the neurons, parvalbumin and calretinin positive GABAergic interneurons also expressed *UBE3A* solely from the maternal allele. Peripheral tissue like liver, heart, and lungs in AS mice showed more than 50% reduction in the levels of *UBE3A* expression, showing that maternal expression was predominant even in the other tissues [69]. 

Further behavioural characterization in this model showed that *UBE*3*A* ^m−/p+^ mice have motor deficits suggestive of a dysfunctional cerebellum [70]. A novel finding was that these mice had a different licking behaviour than the wild-type mice, with more number of licks at greater intervals. It is possible that the difference in the lick behaviour is due to the loss of synchrony between breathing and swallowing and correlates with the feeding and swallowing difficulties seen in AS children [19, 71]. Although the motor deficits observed in *UBE*3*A* ^m−/p+^ mice are thought to be due to dysfunction of cerebellar Purkinje cells, a recent report indicated probable abnormalities in nigrostriatal pathway [33, 72, 73]. The *UBE*3*A* ^m−/p+^ mice showed reduced number of dopaminergic neurons in the substantia nigra accompanied by poor performance in behavioural paradigms sensitive to nigrostriatal dysfunction [74]. This is further supported by the fact that two patients with AS have been shown to manifest typical features of Parkinson's disease like tremors, cogwheel rigidity, and bradykinesia and were responded to levodopa, which is widely used for the symptomatic treatment of Parkinson disease [75]. However, similar disabling tremor in AS patients also has been treated differently [76, 77]. 

Lately, there have been major advancements in understanding the molecular basis of the cognitive deficits associated with AS. The level of the inhibitory phosphorylation at Thr305 of the calcium/calmodulin-dependent protein kinase II (CaMKII) in the hippocampus of the *UBE*3*A* ^m−/p+^ mice was increased leading to reduction in the activity of the protein [36]. The role of CaMKII in the induction of LTP is well established. All the behavioural and learning deficits observed were reversed when a mutation was introduced to block the inhibitory phosphorylation of CaMKII [78]. A very important advancement came with the study of Yashiro et al. [35]. *UBE*3*A* ^m−/p+^ mice were shown to have impaired experience-dependent synaptic plasticity in the visual cortex. Brief monocular deprivation revealed that *UBE*3*A* ^m−/p+^ mice do not show ocular dominance plasticity. This impairment is reversible, and late postnatal deprivation of sensory inputs again restores plasticity of the synapses. These observations suggest that absence of *UBE3A* leads to the inability to modify or rearrange synapses as per the requirement in activity-dependent synaptic plasticity. It is hypothesized that this could occur either due to decreased number of excitatory synapses or due to decreased efficiency of neurotransmitter release. The second probability is in turn dependent on the calcium levels and receptor trafficking which very well correlates to the CaMKII levels. It was observed that the visual cortical circuitry and the retinotopic map are formed normally, but the basal dendrites show reduced spines in *UBE*3*A* ^m−/p+^ mice [37]. This was consistent with the earlier studies [34]. Absence of *UBE3A* plays a crucial role in the postnatal experience driven period [35, 37]. This correlates with the AS patient history of normal birth but delayed developmental milestones. Cognitive development and development of speech are events that depend on the external sensory experience [79]. Failure of these important processes in AS patients could mean that *UBE3A* is indeed required for remodeling of the circuitry. The work so far emphasizes that *UBE3A* is not directly involved in circuit formation but is crucial in experience-dependent synaptic remodeling.

Recently, the exact role of *UBE3A* in experience-driven synaptic plasticity was elucidated at the molecular level [54]. UBE3A mRNA and protein levels are regulated by synaptic activity. UBE3A levels are increased after treatments with kainic acid, KCl, NMDA (N-methyl-D-aspartic acid), glutamate, and bicuculline in primary neuronal cultured cells, while novel environment increases the levels of *UBE3A* in mice brain compared to standard laboratory caged mice. The promoter of *UBE3A* gene is under the control of activity-dependent transcription factor MEF2. The increase in levels of UBE3A with glutamate stimulation and decrease with inhibitors of glutamate receptors clearly puts forth the role of UBE3A in synapse development. Many substrates of UBE3A have been discovered but none were directly implicated in the loss of synaptic plasticity. HA-ubiquitin transgenic mice were crossed with *UBE*3*A* ^m−/p+^ mice, and the proteins that showed reduced ubiquitination were studied. Sacsin was one of the substrates of UBE3A as it showed reduced ubiquitination in knockout mice as compared to wild type. Sacsin is mutated in Charlevoix-Saguenay spastic ataxia, a disorder similar to AS [80]. It is mainly expressed in the neurites of the neurons [81]. The exact role of sacsin in modulation of synapses remains unknown. But sacsin could be one of the causes of the motor deficits seen in AS patients, considering its involvement in disorders with ataxia. Arc was another substrate discovered, which is responsible at least in part for the rigidity seen at the UBE3A deficient synapses. Arc regulates surface expression of AMPARs (alpha-amino-3-hydroxy-5-methyl-4-isoxazole-propionate receptors). Increased Arc expression leads to decreased surface AMPARs while decrease in Arc levels leads to increase in the AMPARs at the surface. Arc promotes the endocytosis of GluA1 type of AMPARs. Lack of *UBE3A* leads to accumulation of Arc, which subsequently results in increased internalization of the AMPARs. UBE3A regulates the surface expression of AMPARs through ubiquitination and proteasomal degradation of Arc. This effect is reduced in presence of catalytically inactive mutants of UBE3A. The decrease in the expression of AMPARs affects the synaptic transmission. There is a reduction seen in the AMPA/NMDA current ratio, which is due to the loss of AMPARs as there was no change in NMDARs. The RhoGEF ephexin5 was also discovered as an UBE3A interacting protein. It has a role in restricting the neuron to form only the required number of synapses [54, 56]. 

Mice expressing UBE3A-YFP fusion protein exclusively from the maternal copy is a very promising tool to carefully study the microscopic abnormalities in AS [34]. Study focusing on the cellular localization of UBE3A helped to elucidate the probable functions of this protein. UBE3A-YFP fusion protein localized mainly in the nucleus with detectable expressions in the cell soma and dendrites. The UBE3A protein was found in the pre and postsynaptic compartments and was localized in the growth cones of hippocampal neurons in primary culture [34, 69]. This mouse model showed biallelic expression of *UBE3A* in GFAP-positive astrocytes lining the ventricular area. In other brain regions GFAP-positive astrocytes seems to exhibit imprinted expression [34]. Although the absence of UBE3A did not affect dendritic branching in any of the imprinted neurons, a detailed microscopic study showed that the dendritic spines had abnormal structures. In the absence of any gross cellular or structural changes in the brain, it is hypothesized that absence of UBE3A is necessary either for the formation or maintenance of the dendritic spines. This is probable since the activity of phospho CaMKII is reduced in maternal deficient animals, and CaMKII is known to help in activity-dependent spine formation. This correlates very well with the observations made in a pathological study in AS brain as well [82]. Further investigation in this mouse model can give major insights into the role of UBE3A during synaptogenesis even at a single synapse level. 

UBE3A is shown to interact with and coactivate nuclear steroid hormone receptors [45, 46, 83]. Absence of UBE3A renders both male and female mice less fertile compared to the wild-type controls [47]. *UBE3A* null male mice show reduced testis size, lesser sperm count, decreased sperm ability to penetrate ova and reduced prostate size. In *UBE3A* knockout female mice, there is reduced oocyte production and smaller ovary size. All these findings indicate that coactivator role of UBE3A is important in reproductive function. But whether the loss of coactivator function of UBE3A is associated with any abnormalities in brain function leading to AS are not very clear. Recently, we have shown that the defective glucocorticoid hormone receptor signaling in *UBE*3*A* ^m−/p+^ mice brain could lead to increased stress and anxiety in these mice. These mice also exhibited decrease in the number of parvalbumin-positive GABAergic interneurons in their hippocampus [84]. 

Yet another mouse model of AS was generated by inactivating the exons corresponding to the human exons 15 and 16 from the *UBE3A* gene [38]. A LacZ reporter was introduced after the deletion site to detect the expressing protein albeit truncated. The expressed UBE3A does not show ligase activity, and the *β*-galactosidase activity is seen in the brain wherever maternal copy expresses the truncated protein. This mouse model showed motor deficits, learning and memory impairments, and an abnormal EEG characteristics of AS, but seizures were absent in this model. UBE3A was imprinted in the hippocampus, basket cells in the cerebellum, as well as in the frontal cortex. Cells in the ventricular ependyma showed LacZ expression both in maternal and paternal *UBE3A* deficient mice, which is consistent with the observation that the ventricular GFAP positive cells express biallelic *UBE3A* [34]. This model confirmed the finding that imprinting is specific to neurons and not astrocytes. Interestingly, it was observed that the progenitor cells do not show imprinted expression, but imprinting is acquired by embryonic day 10 in mouse. Neurons specifically expressed the maternal sense *UBE3A,* while the antisense *UBE3A* was expressed only from the paternal copy [85]. Surprisingly, there was no imprinted expression in the cerebellar Purkinje cells which is a deviation from the other studies [33, 34, 69]. As the protein is truncated only in the C-terminal HECT domain, the transcriptional coactivator function is still might be active in the animals. Absence of imprinted expression in Purkinje cells is a major drawback of the model and could be a reason for unaltered p53 levels. Interestingly, this mouse model showed disrupted sleep wake cycle seen in most of the AS children [2, 86]. Using this mouse model, another group [87] has shown that the deficiency of *UBE3A* leads to impaired neurogenesis and changes in the hippocampal plasticity. The immediate early genes *c-fos* and *arc*, associated with neuronal long-term plasticity and memory formation, showed reduced expression in the maternal deficient mice brain. 

A knockout mouse model of the GABA_A_ (*γ*-amino butyric acid) receptor *β*3 subunit (GABRB3) showed most of the behavioural features like epilepsy, abnormal EEG pattern, learning deficits, and poor motor coordination [88]. Absence of *β*3 subunit leads to neonatal deaths and cleft palates in the animals. The deletions in *GABRB3* are heritable, but since this gene is not imprinted in the brain, *GABRB3* only adds to the phenotypic characteristics and is not a direct cause of AS [89]. Mutation in *UBE3A* is sufficient to show the cardinal features of AS, although deletion of *GABRB3* might contribute to a more severe phenotype [88, 90]. A new mouse model of AS, has been reported recently that tries to replicate the most prevalent form of the syndrome [39]. A 1.6 Mb region spanning from *UBE3A* to *Gabrb3* was deleted to generate this mouse model [39]. Homozygous mutations showed phenotype similar to the *Gabrb3* null mutant. These homozygous null mice showed cleft palate and lethality around the time of birth. The maternal deficient mice of this region, on the other hand, showed no developmental abnormality. They showed spontaneous seizure activity and abnormal EEG. Like the earlier *UBE*3*A* ^m−/p+^ mice, these mice also showed impairment in motor activity and learning and memory. The anxiety related behavior was assessed in these mice and found that maternal deficient mice spent more time in dark areas as compared to the wild-type or paternal deficient mice. Maternal deficient mice with deletion of this region exhibited contextual fear and spatial learning deficits. These mice also showed abnormal pattern of ultrasonic vocalizations [39]. These may correlate with the lack of speech and impaired communication seen in AS patients. 

Another mouse model was generated with an inheritable transgene insertion (Epstein-Barr virus Latent Membrane Protein 2A, *LMP2A*) into the central part of chromosome 7 of mouse [41]. The deletion created by transgene insertion led to formation of either PWS or AS model in a parent-of-origin manner. Inheritance of the deletion from the paternal allele led to formation of PWS, while maternal transmission led to an AS model. *UBE3A* was imprinted in the cerebellum in these mice. Behavioural studies were not reported in this model. Around 70% of the cases in humans are due to deletions in the 15q11-13 region. This model, therefore, represents the widely prevalent condition of AS and, therefore, should be characterized for better understanding of disease pathogenesis and developing therapeutics. Several other mouse model have been generated based on AS imprinting defect mutation [43, 91], radiation-induced mutation removing multiple genes including *UBE3A* [44], and duplication of the AS-PWS locus [42]. Although all of these mouse models reported reduced expression of *UBE3A,* their neurobehavioral phenotype are not well characterized. A list of AS mouse models are shown in Table 2. Interestingly, mice over expressing triple the dose of *UBE3A* showed autism traits like impaired communication, defective social interaction, and increased repetitive stereotypic behavior [92]. These findings along with others [54] clearly indicate that UBE3A plays a very important role in synaptic function, and its altered function could be linked with both AS and autism. In addition to these mouse models, human induced pluripotent stem cell model of AS or mouse differentiated embryonic stem cell model of AS were also developed [93, 94]. These models will be useful to understand the developmental timing and mechanism of *UBE3A* silencing in neurons as well as disease biology.

## 5. Fly Models of AS

*Drosophila* models have also been generated in order to understand the pathogenesis of AS. d*UBE3A*, the homologue of human *UBE3A*, is deleted imprecisely such that the corresponding protein is not formed [95]. Lack of d*UBE3A* is not lethal and the flies born show no morphological abnormality. However, they do show motor abnormalities when tested on motor specific tasks. They have impaired long-term memory formation and abnormal circadian rhythms. Missense mutations analogous to the ones found in AS patients were also used to study their effect. These catalytically inactive mutants show the same behavioral deficits like the d*UBE3A* null flies. Very importantly, this report studies the effect of over expression of d*UBE3A*. The gain-of-function model in this case is particularly informative since the deletion of d*UBE3A* does not lead to any morphological abnormality. Over activity of d*UBE3A* in general is lethal to the flies. Promoter specific expression in the eyes and wings leads to aberrant morphology of the organs. 

Another fly model corroborated the findings of mouse models of the disease [96]. The group studied RNAi d*UBE3A* flies in addition to the deletion mutants. In an interesting approach, they also studied flies by mosaic analysis with a repressible cell marker (MARCM) in which a single neuron is injected with GFP labeled genetic mutation while the surrounding neurons continue to have a wild genotype. Using these advanced techniques, they found that d*UBE3A* is necessary for dendritic arborization in a cell autonomous manner. Absence of d*UBE3A* leads to reduced formation of terminal dendritic branching. Surprisingly, over expression of d*UBE3A* also causes reduction in dendritic branching in the fly, suggesting that the levels of *UBE3A* are critical in formation of the dendrites. The fly, model would be useful in identifying and characterizing the substrates of UBE3A and understanding the disease pathogenesis. 

## 6. Conclusions and Future Perspectives

It is evident from the existing literature that the loss of expression of maternal-inherited *UBE3A* is primarily responsible for AS, although we cannot completely rule out the possibility of other disease-modifying gene like *GABRB3*. Dysfunction of UBE3A is sufficient to produce phenotypes resembling to AS in different animal models. Most extensively used *UBE3A*-maternal deficient mice replicate many essential features of AS including cognitive and motor deficits. This mouse model provided enormous insight in understanding the disease pathogenic mechanism. Clinical features of AS like cognitive and motor deficits, sleep disturbance, feeding difficulties, and altered synaptic plasticity have a molecular or electrophysiological correlate due to the studies performed in animal models. A recent clinical study reported that specific EEG pattern could be an important biomarker of AS and might indicate the underlying genetic cause [18]. This can be further tested in various mouse models to validate the results. Most interestingly, UBE3A-maternal deficient mice show significant impairment in activity-dependent synaptic plasticity indicating the role of UBE3A in regulation of synaptic function and plasticity [54]. The experience-dependent synaptic plasticity is shown to be modulated by number of ways [97]. Therefore, this novel role of UBE3A can be exploited further for possible therapeutic intervention of AS. In fact one report demonstrated neuregulin-ErbB4 signaling is associated with abnormal synaptic plasticity in *UBE*3*A* ^m−/p+^ mice, and inhibitors of ErbB reverse the contextual fear memory deficits [53]. The cognitive deficits observed in *UBE*3*A* ^m−/p+^ mice were also rescued upon adeno-associated virus vector-mediated expression of *UBE3A* into the brain [98]. Since the paternal copy of *UBE3A* is epigenetically silenced in neurons, it is possible that the reactivation of paternal expression could be an exciting therapeutic strategy. Clinical trials were conducted in AS children using methylation-promoting dietary supplements (creatine, folic acid vitamin B12, metafolin, and betaine) in order to up-regulate the *UBE3A* expression (by suppressing the expression of UBE3A antisense transcript). Unfortunately, there were no significant improvements of intellectual disabilities or abnormal EEG patterns in AS children [99, 100]. Interestingly, a very recent report has demonstrated that topoisomerase inhibitors activate the dormant expression of UBE3A in neurons [101]. This is an exciting development. However, treatment of such drugs could also alter the expression of other genes and, therefore, lead to other complications. Further studies are required to investigate possible role of these topoisomerase inhibitors in the recovery of behavioral abnormalities in animal models. Most preferable strategy could be targeted knockdown of the antisense transcript. Enriched environment or neuronal activity (that can trigger experience-dependent synaptic development) also has been demonstrated to increase the expression of *UBE3A *[54]. Therefore, various cognitive training paradigms in early developmental stage could potentially improve cognitive and motor deficits in AS children by increasing the expression of UBE3A. All together, the field is now passing through an exciting phase, and we all are hoping for a major breakthrough in therapeutic intervention of AS.

## Figures and Tables

**Table 1:** Mouse models of AS and their phenotypes.

Animal models	Associated phenotypes
UBE3A^m-/p+^ mice. Deletion of maternal Exon 2 of *UBE3A* [33–37].	Cognitive and motor deficits and inducible seizures. Loss of *UBE3A* expression in neurons, reduced dendritic spine density and defect in hippocampal LTP.
UBE3A^m-/p+^ mice. Deletion of maternal Exons 15 and 16 of *UBE3A* [38].	Cognitive and motor problems, decreased REM sleep, and abnormal EEG, seizures. Loss of *UBE3A* expression in neurons.
DelUBE3A-Gabrb3^m-/p+^ mice. 1.6 Mb maternal deletion disrupting *UBE3A*, *Atp10a*, and *Gabrb3* loci [39].	Increased ultrasonic vocalization, spontaneous seizures, abnormal EEG, impaired learning and memory. Loss of *UBE3A* expression in neurons.
Mice generated with paternal duplication of central region of chromosome 7 (homologous to the human region 15q11-13) [40].	Abnormal EEG, Gait ataxia, abnormal limb clasping, and startle response, hyperactivity. Loss of expression of *UBE3A* in Purkinje cells, hippocampus and olfactory bulb.
Mice created with maternal deletion of central part of chromosome 7 through inheritable transgene insertion [41].	Behavioural abnormalities are not reported. Mice show imprinted expression of *UBE3A* in cerebellum.
Mice created with paternal duplication of chromosome 7 (corresponding to the region of human chromosome 15q11-13) [42].	Abnormal ultrasonic vocalization, poor social interaction, and anxiety. Reduced *UBE3A* expression in brain.
Mice with imprinting defect mutation (corresponding to human AS-IC) [43].	Behavioural phenotypes are not reported. Reduced *UBE3A* expression in brain.
Mice with large radiation-induced deletion of p30PUb [44].	Behavioural phenotypes are not reported.

Number in the brackets indicates references.

**Table 2:** Cellular functions regulated by UBE3A.

Identified substrates	Cellular functions
HHR 23A, Src family tyrosine kinase Blk, P53, P27, PML tumor suppressor [48–52].	Cell growth and differentiation
Steroid hormone receptors like androgen receptor, glucocorticoid receptor, mineralocorticoid receptor [45, 46, 84].	Coactivator of steroid hormone receptors
Arc, RhoA-GEF ephexin5, Rho-GEF Pbl/ECT2 [54–58].	Synaptic function and plasticity
Polyglutamine proteins, *α*-synuclein, misfolded proteins [59–61].	Cellular protein quality control

Number in the brackets indicates references.
